# Derivation and Validation of Two Decision Instruments for Selective Chest CT in Blunt Trauma: A Multicenter Prospective Observational Study (NEXUS Chest CT)

**DOI:** 10.1371/journal.pmed.1001883

**Published:** 2015-10-06

**Authors:** Robert M. Rodriguez, Mark I. Langdorf, Daniel Nishijima, Brigitte M. Baumann, Gregory W. Hendey, Anthony J. Medak, Ali S. Raja, Isabel E. Allen, William R. Mower

**Affiliations:** 1 Department of Emergency Medicine, University of California, San Francisco, California, United States of America; 2 Department of Emergency Medicine, University of California, Irvine, California, United States of America; 3 Department of Emergency Medicine, University of California, Davis, California, United States of America; 4 Department of Emergency Medicine, Cooper Medical School of Rowan University, Camden, New Jersey, United States of America; 5 Department of Emergency Medicine, UCSF Fresno Medical Education and Research, Fresno, California, United States of America; 6 Department of Emergency Medicine, School of Medicine, University of California, San Diego, California, United States of America; 7 Department of Emergency Medicine, Massachusetts General Hospital/Harvard Medical School, Boston, Massachusetts, United States of America; 8 University of California, San Francisco, California, United States of America; 9 Department of Emergency Medicine, University of California, Los Angeles, California, United States of America; Research Center INSERM U897, FRANCE

## Abstract

**Background:**

Unnecessary diagnostic imaging leads to higher costs, longer emergency department stays, and increased patient exposure to ionizing radiation. We sought to prospectively derive and validate two decision instruments (DIs) for selective chest computed tomography (CT) in adult blunt trauma patients.

**Methods and Findings:**

From September 2011 to May 2014, we prospectively enrolled blunt trauma patients over 14 y of age presenting to eight US, urban level 1 trauma centers in this observational study. During the derivation phase, physicians recorded the presence or absence of 14 clinical criteria before viewing chest imaging results. We determined injury outcomes by CT radiology readings and categorized injuries as major or minor according to an expert-panel-derived clinical classification scheme. We then employed recursive partitioning to derive two DIs: Chest CT-All maximized sensitivity for all injuries, and Chest CT-Major maximized sensitivity for only major thoracic injuries (while increasing specificity). In the validation phase, we employed similar methodology to prospectively test the performance of both DIs.

We enrolled 11,477 patients—6,002 patients in the derivation phase and 5,475 patients in the validation phase. The derived Chest CT-All DI consisted of (1) abnormal chest X-ray, (2) rapid deceleration mechanism, (3) distracting injury, (4) chest wall tenderness, (5) sternal tenderness, (6) thoracic spine tenderness, and (7) scapular tenderness. The Chest CT-Major DI had the same criteria without rapid deceleration mechanism. In the validation phase, Chest CT-All had a sensitivity of 99.2% (95% CI 95.4%–100%), a specificity of 20.8% (95% CI 19.2%–22.4%), and a negative predictive value (NPV) of 99.8% (95% CI 98.9%–100%) for major injury, and a sensitivity of 95.4% (95% CI 93.6%–96.9%), a specificity of 25.5% (95% CI 23.5%–27.5%), and a NPV of 93.9% (95% CI 91.5%–95.8%) for either major or minor injury. Chest CT-Major had a sensitivity of 99.2% (95% CI 95.4%–100%), a specificity of 31.7% (95% CI 29.9%–33.5%), and a NPV of 99.9% (95% CI 99.3%–100%) for major injury and a sensitivity of 90.7% (95% CI 88.3%–92.8%), a specificity of 37.9% (95% CI 35.8%–40.1%), and a NPV of 91.8% (95% CI 89.7%–93.6%) for either major or minor injury. Regarding the limitations of our work, some clinicians may disagree with our injury classification and sensitivity thresholds for injury detection.

**Conclusions:**

We prospectively derived and validated two DIs (Chest CT-All and Chest CT-Major) that identify blunt trauma patients with clinically significant thoracic injuries with high sensitivity, allowing for a safe reduction of approximately 25%–37% of unnecessary chest CTs. Trauma evaluation protocols that incorporate these DIs may decrease unnecessary costs and radiation exposure in the disproportionately young trauma population.

## Abstract 2

In this multicenter prospective observational study, Robert Rodriguez and colleagues derive and validate two decision instruments for identifying which blunt trauma patients should receive chest CT and which can avoid unnecessary imaging.

## Editors' Summary

**Background:**

Trauma—a serious injury to the body caused by violence or an accident—is a major global health problem. Every year, events that include traffic collisions, falls, and burns cause injuries that kill more than 5 million people (9% of annual global deaths). Road traffic accidents alone cause about 1.24 million deaths per year. In many countries, including the US, trauma is the number one killer of individuals aged 1–46 years. Chest injuries—damage to the chest wall such, as rib fractures, or damage to the lungs, heart, airways, or major blood vessels within the chest—are responsible for a quarter of trauma deaths. Chest injuries can be penetrating or blunt. Penetrating injuries (for example, stabbings) are generally easy to diagnose and usually require surgery. Blunt injuries, which are often the result of falls or road accidents, can often be managed with relatively simple interventions such as mechanical ventilation but can be hard to diagnose.

**Why Was This Study Done?:**

Computed tomography (CT) is often used to evaluate patients with blunt trauma. This imaging procedure uses special X-ray equipment and computer programs to create two-dimensional and three-dimensional pictures of the organs, bones, and other tissues of the body. CT is good at providing information about internal injuries, and many trauma centers now routinely examine victims of major trauma using head-to-pelvis CT. However, chest CT exposes patients to radiation doses that may increase their risk of cancer. Moreover, chest CT is expensive and does not always provide much additional information if completed after a normal chest X-ray. To reduce the costs and radiation risks of unnecessary CT imaging after blunt trauma, in this prospective observational study, the researchers develop and validate two clinical decision instruments that identify patients with blunt chest injuries, thereby allowing clinicians to forego CT in patients who do not meet the decision instrument criteria for blunt injuries.

**What Did the Researchers Do and Find?:**

The researchers had clinicians record the presence or absence of 14 candidate clinical criteria in 6,002 patients aged over 14 years attending eight US trauma centers with blunt trauma before viewing chest CT results and categorizing the injuries seen as major or minor using a preset classification scheme. They then derived two clinical decision instruments from these data using a statistical method called recursive partitioning. The Chest CT-All decision instrument, which maximized sensitivity (the ability to correctly identify people with a condition) for either major and minor chest injuries, consisted of seven clinical criteria including an abnormal x-ray, rapid deceleration mechanism (trauma caused by, for example, a road collision occurring at more than 40 mph), and bone tenderness (pain that occurs when an area is touched) in the chest. The Chest CT-Major instrument, which maximized sensitivity for only major chest injuries, consisted of the same criteria without rapid deceleration mechanism. Applied to 5,475 additional patients who presented with blunt trauma, the Chest CT-All instrument correctly identified 95.4% of patients with a major or minor blunt chest injury as having an injury (a sensitivity of 95.4%) and 25.5% of patients without a major or minor injury as not having an injury (a specificity of 25.5%); the instrument had a negative predictive value (NPV) of 93.9% (a patient judged injury-free using the instrument had a 93.9% probability of being injury-free). The Chest CT-Major instrument had a sensitivity of 99.2%, specificity of 31.7%, and NPV of 99.9% for major injuries.

**What Do These Findings Mean?:**

These findings describe two decision instruments that detect clinically important blunt chest injuries with high sensitivity. Because the use of these instruments allows clinicians to identify virtually everyone who has this type of injury, clinicians can forego CT in patients who do not exhibit any of the decision instrument criteria for blunt chest injury. That is, clinicians can safely use physical examination and history findings, instead of imaging, to rule out blunt chest injury in many patients attending a trauma center. Limitations of this study include the criteria used to classify injuries as minor or major. Moreover, these decision instruments should be used to augment rather than replace clinical judgment and should not be used to evaluate patients younger than 15 years old. Importantly, however, use of these decision instruments could reduce the number of unnecessary chest CTs undertaken in trauma centers by up to a third, thus reducing costs and radiation exposure in people with trauma.

**Additional Information.:**

This list of resources contains links that can be accessed when viewing the PDF on a device or via the online version of the article at http://dx.doi.org/10.1371/journal.pmed.1001883.

- This study is further discussed in a *PLOS Medicine* Perspective by Emmanuel Lagarde
- The World Health Organization provides information in several languages about injuries around the world; its Guidelines for Essential Trauma Care seeks to set achievable standards for trauma treatment services that could realistically be made available to almost every injured person in the world
- The US National Institute of General Medical Sciences has a factsheet on trauma
- The National Trauma Institute, a not-for-profit organization that supports research on trauma, provides US trauma statistics, trauma survivor stories, and links to other organizations in the US that provide information on trauma
- Wikipedia has pages on major trauma, chest injury, and computed tomography (note that Wikipedia is a free online encyclopedia that anyone can edit; available in several languages)
- MedlinePlus provides links to additional information about trauma and about chest injuries and disorders (in English and Spanish)

## Introduction

The use of computed tomography (CT) in adult blunt trauma evaluation has risen dramatically in the past two decades, and many trauma centers have adopted routine head-to-pelvis CT protocols (pan-scan) that include chest CT for victims of major trauma [1–7]. Multiple investigators have concluded, however, that this escalation in CT use is associated with clear and quantifiable cancer risks [8–11]. Several major specialty organizations have therefore called for a review of widespread CT use in trauma, and in 2014 the American College of Surgeons listed avoidance of routine whole body trauma CT as one of its five Choosing Wisely recommendations [12–14].

With an effective radiation dose among the highest in all diagnostic imaging, the cancer induction risk of chest CT is considerable [7]. Unnecessary trauma chest CT may also be especially expensive. In our previous blunt trauma work, we showed that chest CT after a normal chest X-ray (CXR) may be a particularly low-yield, concerning practice, resulting in over US$200,000 in hospital charges and potentially inducing one cancer for every 23 major injuries diagnosed [15].

Seeking to reduce the costs and radiation risks of unnecessary blunt trauma imaging, our objective in this study was to derive and validate clinical decision instruments (DIs) that identify patients with thoracic injury and can therefore safely guide selective ordering of chest CT by allowing clinicians to forego CT in patients who do not have any DI criteria. Recognizing that practice patterns vary among clinicians, we sought to establish two DIs: the first (Chest CT-All) would have high sensitivity for all thoracic injuries seen on chest CT, and the second (Chest CT-Major) would have high sensitivity for major injuries, while accepting a small miss rate for minor injuries (in order to maximize specificity).

## Methods

### Sites, Participants, and Enrollment

We conducted this study at eight urban, US level 1 trauma centers located in California (San Francisco, Irvine, Los Angeles, Davis, Fresno, and San Diego), New Jersey (Camden), and Massachusetts (Boston). We conducted the derivation phase from September 2011 to December 2012 and the validation phase from February 2013 to May 2014. Our inclusion criteria in both phases of the study were as follows: (1) being over 14 y of age, (2) presenting to the emergency department (ED) for blunt trauma that occurred within 6 h of arrival, and (3) having chest imaging (either CXR or chest CT) ordered in the ED (including trauma resuscitation rooms). Using convenience sampling primarily from 07:00 to 23:00, research personnel enrolled patients when CXR or chest CT was ordered. We excluded patients after enrollment if we later determined that they did not receive chest imaging, were under 15 y of age, or primarily had a penetrating (e.g., gunshot wound, stabbing) mechanism of trauma. We left all imaging decisions to the discretion of the treating providers.

### Decision Instrument Candidate Criteria

From our prior NEXUS Chest study work, reviews of the literature, and investigator consensus, we generated a list of 14 candidate DI criteria [16–19]: (1) age greater than 60 y, (2) altered mental status or altered level of consciousness, (3) intoxication, (4) rapid deceleration mechanism, (5) chest pain, (6) distracting injury, (7) chest wall tenderness, (8) sternal tenderness, (9) thoracic spine tenderness, (10) scapular tenderness, (11) abnormality in the pericardial window of focused assessment with sonography in trauma (FAST), (12) abnormality in any of the abdominal FAST windows, (13) abnormality in the pulmonary component of extended FAST (eFAST), and (14) abnormality in portable CXR. We had previously determined that shortness of breath, abnormal chest auscultation, visible chest wall skin injury, and hypoxia (oxygen saturation on ED presentation of <95%) had insufficient discriminatory value for use as DI criteria [16]. We presented the candidate criteria on data collection forms, along with definitions (see S1 Appendix), to trauma providers and asked them to record the presence or absence of these criteria prior to their review of any chest imaging results.

To measure inter-rater reliability of clinical criteria, we conducted dual, independent provider criteria assessments of 160 patients (20 from each study site) and calculated a kappa statistic of agreement. Only criteria with a kappa > 0.6 were considered acceptable for use in the DIs. To avoid overestimation of the safety (sensitivity) of the DIs, missing and unknown criteria (<0.1% of the total) were assumed to be absent.

### Outcomes

Prior to the study, we generated a comprehensive list of thoracic and intra-thoracic injuries seen on thoracic CT. We then convened an expert panel of ten physicians at the associate professor level or higher (six emergency medicine physicians and four trauma surgeons) to classify these injuries as clinically major, minor, or insignificant based on associated interventions, observation, and hospital admission. See Table 1 for this classification.

**Table 1:** Trauma expert panel determination of clinical significance of injuries seen on chest imaging.

Category	Injury
**Major clinical significance**	Aortic or great vessel injury (all considered major)
	Ruptured diaphragm (all considered major)
	Pneumothorax: received evacuation procedure (chest tube or other procedure)
	Hemothorax: received drainage procedure (chest tube or other procedure)
	Sternal fracture: received surgical intervention
	Multiple rib fracture: received surgical intervention or epidural nerve block
	Pulmonary contusion: received mechanical ventilation (including non-invasive ventilation) primarily for respiratory failure within 24 h for management
	Thoracic spine fracture: received surgical intervention
	Scapular fracture: received surgical intervention
	Mediastinal or pericardial hematoma: received drainage procedure
	Esophageal injury: received surgical intervention
	Tracheal or bronchial injury: received surgical intervention
**Minor clinical significance**	Pneumothorax: no evacuation procedure but observed as inpatient >24 h
	Hemothorax: no drainage procedure but observed as inpatient for >24 h
	Sternal fracture: no surgical intervention
	Multiple rib fracture: no surgical intervention or epidural nerve block
	Pulmonary contusion or laceration: no mechanical ventilation but observed >24 h
	Thoracic spine fracture: no surgical intervention
	Scapular fracture: no surgical intervention
	Mediastinal or pericardial hematoma: no surgical intervention
	Esophageal injury: no surgical intervention
	Tracheal or bronchial injury: no surgical intervention
**No clinical significance** *	Hemothorax: no surgical intervention, no inpatient observation
	Pneumothorax: no surgical intervention, no inpatient observation
	Pneumomediastinum without pneumothorax: no inpatient observation
	Pulmonary contusion or laceration: no mechanical ventilation, no surgical intervention, no inpatient observation

*This category was generated to account for those instances in which CT visualizes minute abnormalities that result in no changes in management.

We used radiologists’ official reports for all imaging outcomes. When radiology reports regarding outcomes were vague (“possible pulmonary contusion”), we assumed the finding to be present. Abnormal CXR was defined as having any thoracic injury (including clavicle fracture) or a widened mediastinum.

Blinded to clinical assessment or DI criteria, study personnel (abstractors) followed all enrolled patients through their hospital course to determine clinical outcomes (hospital admission and injury-associated interventions). In order to check abstractor reliability, a second abstractor independently abstracted patient outcomes (radiology reports, admission, and interventions) in 80 of the first 1,000 patients. Given that we found extremely high inter-abstractor agreement for all outcomes (radiology reports—99% agreement, kappa = 0.97; hospital admission and interventions—100% agreement, kappa = 1.0), we limited our subsequent checks of outcomes to random monthly audits.

### Sample Size Considerations and Analyses

The explicit goal of this work was to develop DIs that clinicians can use to effectively rule out injury and forego imaging in patients who do not have any DI criteria. Because sensitivity is less affected by prevalence variations than negative predictive value (NPV), we focused on sensitivity and calculated our sample size based on the need to derive and validate DIs that were highly sensitive for clinically significant injury. We sought a 95% CI width of approximately 2% around the targeted sensitivities of 98% for major injury and 95% for major or minor injury. Using lower end estimates of the prevalence of injury in patients who had CXR and CT at an early point of data collection in our prior work (5% for major injury and 10% for major or minor injury) [17], we calculated that we would need to enroll at least 4,570 patients in each phase of the study. We continued enrollment beyond this sample size in both phases as a buffer for variations in prevalence of injury.

We used binary recursive partitioning in R (algorithms BRP and Rpart, R version 3.1.1 [2014]) to derive Chest CT-All and Chest CT-Major. We sought to maximize sensitivity for clinically major injury, with a goal of 99%, by omitting candidate criteria and recalculating sensitivity and DI stability systematically via a jackknife analysis. For Chest CT-All we additionally sought a sensitivity for major or minor injury of at least 95%. After we derived Chest CT-All, we derived the higher specificity Chest CT-Major by removing criteria, with the same goal of high sensitivity for major injury but a lower acceptable sensitivity for major and minor injury of at least 90%.

We used Research Electronic Data Capture (REDCap), hosted by the University of California, San Francisco, to manage data [20]. After validation phase data collection, we calculated the relevant screening performance parameters (sensitivity, specificity, NPV, and negative likelihood ratio) of Chest CT-All and Chest CT-Major using Stata v. 13.2 (StataCorp).

### Controls for Bias

Our explicit goal was to develop DIs that detect clinically significant injuries seen on chest CT performed in the ED. In order to control for the potential spectrum bias that could arise from the fact that less than half of trauma patients receive chest CT, we enrolled and followed all patients who received any ED chest imaging even if they did not receive chest CT. We performed a sensitivity (and full screening performance) analysis of our DIs in this larger group of patients taken from the validation cohort, comparing DI performance in this population to the DI performance in the primary analysis (the subgroup of patients who received both CXR and chest CT). Regarding bias that could arise from lack of enrollment of patients who did not receive any imaging at all in the ED, we have previously demonstrated that the clinically significant injury rate in this non-imaged group of patients approaches zero and is therefore negligible [17].

Using a multi-campus review mechanism, we obtained institutional review board approval from the University of California San Francisco Committee on Human Research for the six University of California sites. We obtained separate approval from the Cooper Medical School of Rowan University and Brigham and Women’s Hospital institutional review boards for their respective participating institutions. Because of the strictly observational nature of the study and the fact that many patients who were critically injured, were intoxicated, or had head injury would be unable to provide consent, we obtained a waiver of consent at all sites. We controlled all aspects of study design, implementation, analysis, and manuscript preparation without influence from the grant funding agency.

## Results

The median age of the 11,477 enrolled patients was 46 y (interquartile range [IQR] 29–62), 61.0% were male, 47.9% were admitted to the hospital, and their median injury severity score was 5 (IQR 1–10). The most common trauma mechanisms were motorized vehicle collisions (35.6%) and falls (27.4%). See Table 2 for demographics separated according to study phase.

**Table 2:** Patient characteristics.

Characteristic	Derivation Phase (*n* = 6,002)	Validation Phase (*n* = 5,475)
**Male gender: number (percent)**	3,583 (59.7)	3,384 (61.8)
**Age: median (IQR)**	46 (29–62)	45 (28–61)
**Mechanism of injury: number (percent)**		
MVA	2,141 (35.7)	1,945 (35.5)
Fall	1,781 (29.7)	1,368 (25.0)
MCA	466 (7.8)	594 (10.8)
PMV	498 (8.3)	543 (9.9)
**GCS: median (IQR)**	15 (14–15)	15 (14–15)
**Admitted to hospital: number (percent)**	2,768 (46.1)	2,733 (49.9)
**Survived to hospital discharge if admitted: number (percent)**	2,599 (93.9)	2,575 (94.2)
**Hospital LOS in days: median (IQR)**	3 (1–5)	3 (1–5)
**ISS: median (IQR)** *	5 (1–10)	5 (1–10)

**Fig 1:**
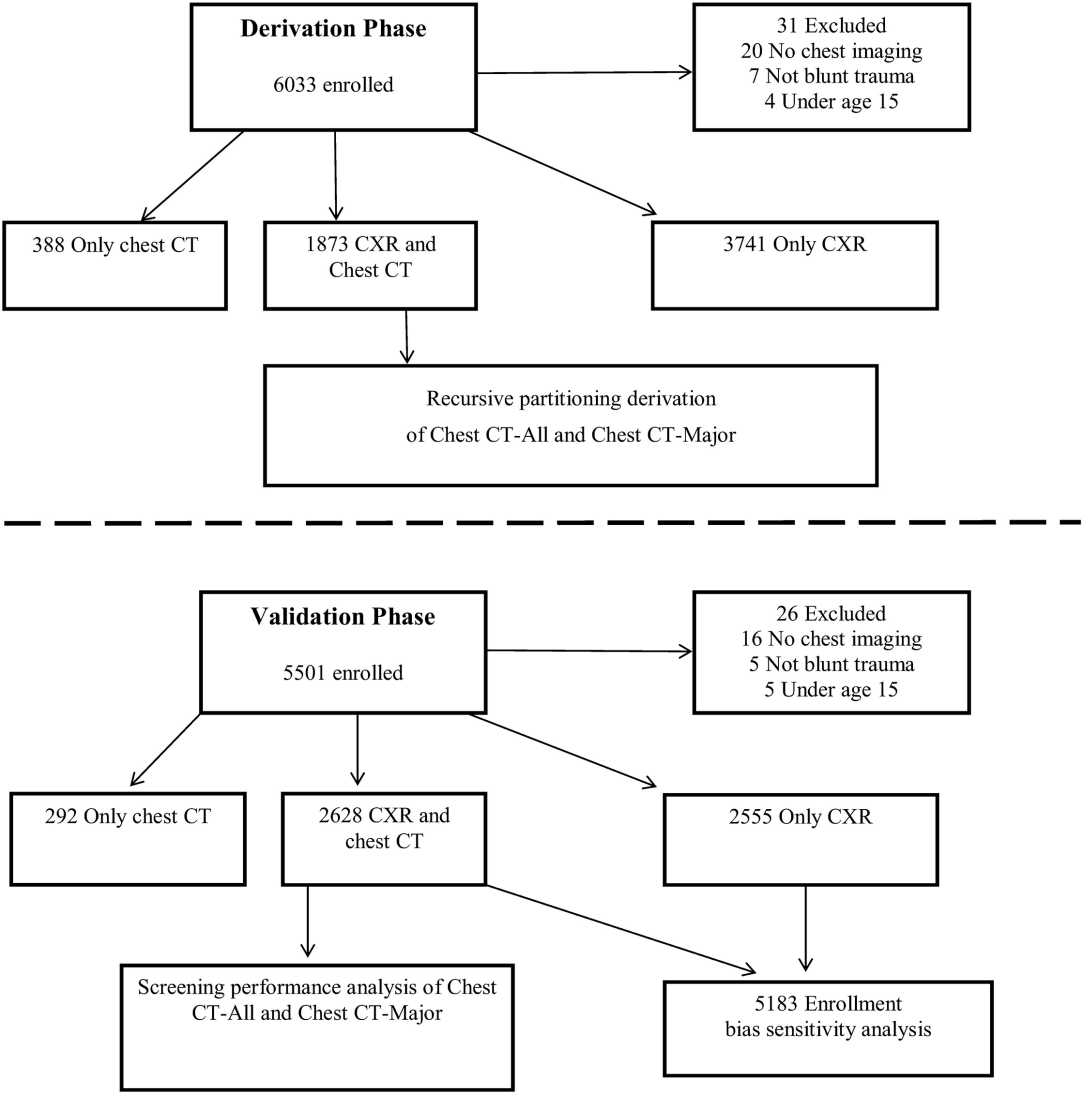
Study flow: derivation and validation phases. In the derivation phase, we enrolled 6,033 patients and derived the DIs on the 1,873 patients who received both CXR and chest CT. We then enrolled 5,501 patients in the validation phase and validated the two DIs in the 2,628 patients who had both CXR and chest CT. Along with these 2,628 patients, we incorporated the 2,555 validation phase patients who received only CXR into an enrollment bias sensitivity analysis.

See Fig 1 for study enrollment.

*Injury severity score assessment was performed in 8,152/11,477 patients—other patients had no (or very minor) injuries and were discharged from the ED.

GCS, Glasgow Coma Scale; ISS, injury severity score; LOS, length of stay; MCA, motorcycle accident; MVA, motorized vehicle accident; PMV, pedestrian struck by motorized vehicle.

Of the 6,002 enrolled and included patients during the derivation phase, 1,873 (31.2%) had both CXR and chest CT; 144 (7.7%) of these patients had clinically major injury and 649 (34.7%) had either clinically major or minor injury. See Tables 3 and 4 for the lists of major and minor injuries diagnosed during both phases.

**Table 3:** Clinically major injuries.

Injury	Derivation Phase (187 Injuries in 144 Patients)	Validation Phase (173 Injuries in 120 Patients)
Pneumothorax: received chest tube	95	90
Hemothorax: received chest tube	33	37
Spinal fracture: received surgical stabilization	17	12
Pulmonary contusion: received mechanical ventilation	11	13
Spinal fracture: received surgical stabilization	9	1
Aortic or great vessel injury: no surgery but observed >24 h	6	5
Aortic or great vessel injury: underwent surgery	5	5
Other thoracic injury: received surgical intervention	4	2
Sternal fracture: received surgical intervention	2	1
Scapular fracture: received surgical intervention	2	2
Mediastinal or pericardial hematoma: received drainage procedure	2	1
Ruptured diaphragm	1	3
Bronchial injury: received surgical intervention	0	1

**Table 4:** Clinically minor injuries.

Injury	Derivation Phase (1,109 Injuries in 633 Patients)	Validation Phase (1,155 Injuries in 691 Patients)
Multiple rib fracture: no surgical intervention or epidural nerve block	407	446
Pulmonary contusion: no mechanical ventilation but observed >24 h	175	236
Spinal fracture: no surgical intervention	123	77
Pneumothorax: no chest tube but observed >24 h	112	126
Sternal fracture: no surgical intervention	110	123
Scapular fracture: no surgical intervention	66	64
Hemothorax: no chest tube but observed >24 h	42	23
Pneumomediastinum without pneumothorax: no surgical intervention but observed >24 h	33	20
Mediastinal hematoma: no surgical intervention but observed >24 h	29	36
Other minor thoracic injury	9	0
Pericardial hematoma: no pericardiocentesis or surgical intervention but observed >24 h	3	3
Esophageal injury: no surgical intervention but observed >24 h	0	1

Because pericardial FAST windows, abdominal FAST windows, and eFAST were reported in only 62%, 63%, and 37% of patients, respectively, they were not included as candidate criteria in our derivation algorithms. Kappa statistics of agreement for the remaining criteria were all acceptable according to our predetermined criteria: distracting injury, 0.60; rapid deceleration mechanism, 0.66; intoxication, 0.79; altered mental status, 0.73; chest pain, 0.75; chest wall tenderness, 0.75; thoracic spine tenderness, 0.78; sternal tenderness; and scapular tenderness, 0.82.

Recursive partitioning produced Chest CT-All, which consisted of (1) abnormal CXR, (2) rapid deceleration mechanism, (3) distracting injury, (4) chest wall tenderness, (5) sternal tenderness, (6) thoracic spine tenderness, and (7) scapular tenderness, and which had a sensitivity for major injury of 99.3% (95% CI 96.2%–100%) and a sensitivity for major or minor injury of 98.2% (95% CI 96.8%–99%). The derived Chest CT-Major DI consisted of the same criteria without rapid deceleration mechanism and had sensitivities for major injury and for major or minor injury of 99.3% (95% CI 96.2%–100%) and 94.8% (95% CI 92.8%–96.3%), respectively.

Of the 5,475 enrolled and included patients during the validation phase, 2,628 (48.0%) had both CXR and chest CT; 120 (4.6%) of these patients had clinically major injury, and 706 (26.9%) had either clinically major or minor injury. Chest CT-All had sensitivities for major injury and for major or minor injury of 99.2% (95% CI 95.4%–100%) and 95.4% (95% CI 93.6%–96.9%), respectively. Chest CT-Major had sensitivities for major injury and for major or minor injury of 99.2% (95% CI 95.4%–100%) and 90.7% (95% CI 88.3%–92.8%), respectively.

Chest CT-All had a NPV for major injury and for major or minor injury of 99.8% (95% CI 98.9%–100%) and 93.9% (95% CI, 91.5%–95.8%), respectively. Chest CT-Major had a NPV for major injury and for major or minor injury of 99.9% (95% CI 99.3%–100%) and 91.8% (95% CI 89.7%–93.6%), respectively.

With a specificity for major or minor injury of 25.5% (95% CI 23.5%–27.5%), Chest CT-All would correctly avoid approximately 25% of non-diagnostic chest CTs. Chest CT-Major would correctly avoid approximately 37% of chest CTs (specificity for major or minor injury = 37.9%, 95% CI 35.8%–40.1%). See Table 5 for the full validation screening performance of Chest CT-All and Chest CT-Major.

**Table 5:** Screening performance characteristics of Chest CT-All and Chest CT-Major in validation cohort (*n* = 2,628).

DI	Injury	Sensitivity	Specificity	Negative Predictive Value	Negative Likelihood Ratio	Positive Predictive Value	Positive Likelihood Ratio
**Chest CT-All**	Major injury (TP = 117, TN = 522, FP = 1,988, FN = 1)	99.2 (95.4–100)	20.8 (19.2–22.4)	99.8 (98.9–100)	0.04 (0.06–0.29)	5.6 (4.6–6.6)	1.3 (1.2–1.3)
	Major or minor injury (TP = 669, TN = 491, FP = 1,436, FN = 32)	95.4 (93.6–96.9)	25.5 (23.5–27.5)	93.9 (91.5–95.8)	0.18 (0.13–0.25)	31.9 (29.8–33.8)	1.3 (1.2–1.3)
**Chest CT-Major**	Major injury (TP = 117, TN = 795, FP = 1,715, FN = 1)	99.2 (95.4–100)	31.7 (29.9–33.5)	99.9 (99.3–100)	0.03 (0.04–0.19)	3.4 (5.3–7.6)	1.5 (1.4–1.5)
	Major or minor injury (TP = 636, TN = 731, FP = 1,196, FN = 65)	90.7 (88.3–92.8)	37.9 (35.8–40.1)	91.8 (89.7–93.6)	0.24 (0.19–0.31)	34.7 (32.5–36.9)	1.5 (1.4–1.5)

Data given as percent (95% CI).

FN, false negative (absence of all DI criteria and having injury); FP, false positive (presence of one or more DI criteria and not having injury); TN, true negative (absence of all DI criteria and not having injury); TP, true positive (presence of one or more DI criteria and having injury).

The sensitivity for major injury of both Chest CT-All and Chest CT-Major in our spectrum bias control group (all patients in the validation cohort who received chest imaging) was 99.2% (95% CI 95.8%–100%). The sensitivities for major or minor injury of Chest CT-All and Chest CT-Major in this control group were 95.8% (95% CI 94.1%–97.1%) and 91.5% (95% CI 89.3%–93.4%), respectively.

Chest CT-All and Chest CT-Major failed to detect one of the 120 patients with a clinically major injury. This 80-y-old male fell down seven stairs and had a subarachnoid hemorrhage and an isolated pneumothorax that was treated with a chest tube. Chest CT-All and Chest CT-Major failed to detect 31 and 64 patients with clinically minor injury, respectively. These minor injuries, by definition, were all non-operative and consisted of rib fractures (25), sternal fracture (8), pulmonary contusion (6), thoracic spine fracture (4), scapular fracture (2), pneumothorax (1), and more than one minor injury (19). All admitted false negative patients survived to hospital discharge. Nine of the 65 (13.4%) false negative patients were discharged to home from the ED, and none of them returned to their index hospital for care within two weeks.

Regarding our assessment of patients who had negative (no injury seen) thoracic imaging in the ED, only one patient in both the derivation and validation cohorts was later diagnosed with a significant thoracic injury (a pneumothorax on hospital day two). Because he was assessed with the criterion of chest wall tenderness, his injury nevertheless would have been picked up by both DIs.

## Discussion

Although trauma chest CT clearly plays an integral role in blunt trauma evaluation, its indiscriminate use results in undeniable risks and costs. Toward the goal of reducing unnecessary trauma chest CT, we developed two DIs that detect clinically significant injuries with very high sensitivity, allowing clinicians to forego CT in patients who do not have DI criteria. In effect, our work provides clinicians with an evidence-based mechanism to use basic physical exam and history findings—instead of advanced imaging—to safely and efficiently rule out injury in appropriate patients.

Our DIs are not the first to attempt to decrease the use of chest CT in blunt trauma patients. In a single site study, Brink et al. derived a blunt trauma chest CT DI with 95% sensitivity for clinically significant injury [18]. However, because this DI requires the performance of several types of imaging beyond CXR (abdominal ultrasound, thoracic spine X-ray, and pelvic X-ray), it is unlikely to be useful to guide acute trauma imaging decisions. Aside from the CXR, all of the criteria in our DIs are simple, straightforward portions of the routine trauma history and physical exam (primarily palpation for tenderness of the bony elements of the thorax and upper back), none of which should require extra provider time, costs, or effort. Foregoing the chest CT portion of a pan-scan has been considered by other trauma investigators. Barrios et al. reported that the addition of thoracic CT in patients who had a normal CXR and abdominal CT was of limited diagnostic utility [19].

We followed the guidelines put forth by Stiell et al. [21] for optimal development of DIs and by Gilbert et al. [22] for chart abstraction. Specifically, we emphasized the use of simple criteria that can be easily and reliably assessed; we controlled extensively for spectrum bias and ensured complete follow-up of all enrolled participants; we defined outcomes independently of the criteria, with complete blinding of abstractors for these elements; and, most importantly, we conducted all of this work prospectively, with separate, independent cohorts for derivation and subsequent validation.

To further optimize the external validity of our work, we emphasized real-world conditions [23]. Our criteria assessments were performed during routine trauma physical exams and recorded before imaging result acquisition, thereby closely replicating the manner in which clinicians would apply a DI for CT decisions.

**Fig 2:**
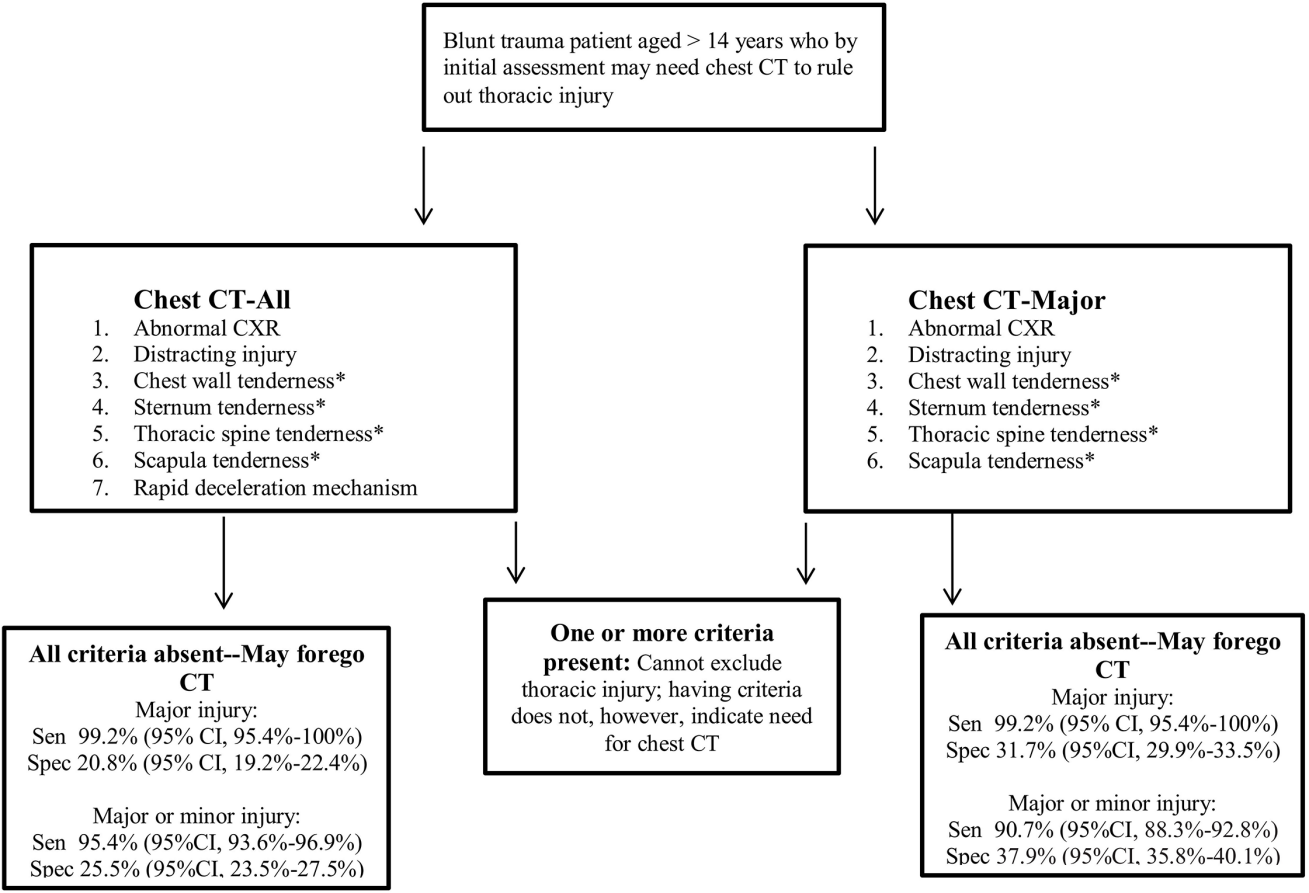
NEXUS Chest CT decision instrument implementation. Abnormal CXR is any thoracic injury (including clavicle fracture) or a widened mediastinum. Rapid deceleration mechanism is a fall from >20 feet (6.1 m) or a motor vehicle accident at >40 miles (64.4 km) per hour with sudden deceleration. Thoracic injury is defined as pneumothorax, hemothorax, aortic or great vessel injury, multiple rib fractures, ruptured diaphragm, sternal fracture, scapular fracture, thoracic spine fracture, esophageal injury, tracheal/bronchial injury, or pulmonary contusion/laceration. *These four criteria may be evaluated together as any thoracic wall, sternal, spine, or scapular tenderness. Sen, sensitivity; Spec, specificity.

When designing this study, we were cognizant of distinct trauma practice variations and ideological differences in terms of injury diagnosis thresholds [24]. We therefore convened a multidisciplinary panel to classify injuries seen on CT and developed two distinct DIs. For clinicians who believe it is important to diagnose all (or nearly all) injuries, we developed Chest CT-All, which detects both major and minor injuries with high sensitivity and which may allow providers to forego unnecessary chest CT in approximately 25% of patients. For clinicians who seek to detect only those injuries that result in interventions, we recommend our second DI, Chest CT-Major, which maintains an equally high sensitivity for major injuries and has a greater specificity of over 37%, thereby sparing more patients from unnecessary CT. The only difference between the two DIs is the inclusion of the criterion of rapid decelerating mechanism in Chest CT-All, which allowed for greater detection of minor injuries. See Fig 2 for our recommended implementation of Chest CT-All and Chest CT-Major.

Vigilance for highly lethal aortic or great vessel injuries is commonly used as a primary justification for ordering chest CT in blunt trauma cases [4–6,25,26]. Our DIs detected all of the 21 aortic/great vessel injuries seen in our derivation and validation sets, and they would have detected all 17 of the aortic injuries in our prior NEXUS Chest studies [16,18]. Notably, CXR was normal (including normal mediastinal width) in eight (38%) of these cases, and there was no rapid deceleration mechanism in seven (33%) of these cases.

FAST, especially eFAST for pneumothorax, has been shown to have great utility in trauma evaluation [27]. Although adherence to our a priori rules of DI development precluded the use of eFAST in our current DIs, we plan to incorporate it in another decision rule.

Perhaps the greatest criticism of our work may come from those who strongly espouse chest CT as part of universal head-to-pelvis CT (pan-scan) in major blunt trauma evaluation [4–6]. We recognize that pan-scan may be the most practical and appropriate diagnostic approach in patients with severe, hemodynamically unstable trauma affecting multiple anatomic regions, especially when clinicians have concerns about repeat CT scans and multiple doses of intravenous contrast [6,7,28,29]. Our DIs, however, are intended to benefit the less critically injured patient, in whom portable anteroposterior CXR is the most common initial diagnostic step. Even the staunchest trauma CT scan advocates may recognize a tipping point for the utility of pan-scan—that there must exist a group of patients in whom the risks (and costs) of CT outweigh its potential diagnostic benefits. With a risk of significant missed injury of approximately 1%, we believe that our DIs meet a practical threshold for safety.

Some authorities may also contend that that the true added cost of CT to hospitals is minimal and that CT radiation concerns are overblown and should not be considered in acute trauma situations [6]. However, patient-centered care requires that physicians consider patients’ views and respect their autonomy whenever possible. We have demonstrated that most patients wish to discuss the risks and costs of trauma CT and that they would often choose to accept a low risk (<2%) of missed life-threatening injury to avoid the radiation exposure and charges attendant with chest CT [30].

The radiation risks of CT are underappreciated, and as we acknowledged and addressed from the outset of our preliminary study planning, opinions vary widely regarding the need to diagnose non-interventional injuries [4–7,31–33]. Prior to the widespread use of chest CT in trauma, minor injuries would commonly go undetected, and providers would focus on dealing with clinical complications as they arose, rather than attempting to diagnose these injuries preemptively. Most of the missed injuries were rib fractures, isolated sternal fractures, and small pulmonary contusions. Most authorities agree that isolated sternal fractures can be managed on an outpatient basis, and, similarly, occult pulmonary contusions (those that are seen only on CT) are of little consequence [34–36]. Given that the management of all these injuries is primarily pain control and observation (care that is routine and occurs with thoracic trauma in general) [37], we believe that the extra radiation exposure and cost of chest CT for a 100% detection rate of these injuries is not justified.

Our work does not refute studies reporting that CXR alone has a low sensitivity for important findings that are seen on chest CT [38–40]. CXR (without the other elements in our DIs) failed to detect many of the injuries in Table 4. Similarly, we are not arguing against the use of CT to better characterize injuries suggested by CXR.

As we have emphasized in our prior work [18], these NEXUS Chest CT DIs are one-way directive rules that should be used only to rule out major injury and forego chest CT. Having DI criteria, such as an abnormal CXR or chest wall tenderness, in no way indicates the need for CT—misapplication of our DIs in this manner can paradoxically lead to greater imaging.

We recognize that clinicians may order CT scans in response to fear of missing injuries and medical-legal concerns. We believe that clinicians, by documenting that patients are low risk based on NEXUS Chest CT DI criteria, may use our work to counter this legal risk in a manner similar to the current clinical implementation of the NEXUS cervical spine and PECARN pediatric head trauma rules [41,42].

### Limitations

Variance in injury prevalence may impact the NPV of our DIs when applied to different populations. Lower level trauma centers likely treat a less severely injured population, and our NEXUS Chest CT DIs may therefore exhibit lower NPV at these sites. Sensitivity, however, should remain consistent.

Although most of the providers assessing patients were resident physicians, potentially limiting external applicability to non-teaching hospitals, all of our criteria are simple, with high inter-rater reliability. We believe that they can be assessed easily by a broad range of clinicians.

We believe that the differences in the rates of major and minor injury diagnosis between the derivation and validation cohorts resulted from changes in CT evaluation protocols at our trauma centers over time. As new trauma protocols dictated that more patients receive automatic head-to-pelvis CT (regardless of whether or not they had evidence of thoracic trauma), the diagnostic efficiency of chest CT was decreased.

With regard to injuries missed by the DIs, most were rib, sternal, thoracic spine, or scapular fractures. Considering that our DIs include criteria for tenderness to palpation of all of these bony structures, these missed injuries were likely very minor (causing no clinically detectable tenderness). It is also possible that some clinicians in the study did not thoroughly examine these structures or that they merely checked boxes when filling out the DI data collection forms; some of these cases may therefore not reflect true injury detection failures.

Practitioners may disagree with our injury classifications, placing greater or lesser value on certain diagnoses. For example, some clinicians may believe that all sternal fractures are major injuries, impacting patient care even without surgical intervention. Our designation of injuries as major and minor, however, reflects a balanced consensus of both emergency medicine physicians and trauma surgeons. Furthermore, our development of two distinct DIs reflects our recognition that perspectives and practice patterns are likely to vary among clinicians.

Finally, our DIs cannot replace clinical judgment—rather they are meant to augment it. They also do not apply to the pediatric population under 15 y of age. Given that the radiation risks of chest CT are higher in younger patients and may be negligible in elderly patients, providers may wish to apply our DIs differentially by age.

### Conclusions

Evaluating two cohorts of adult blunt trauma patients, we prospectively derived and validated two NEXUS Chest CT DIs, which consist of simple, readily available criteria that detect clinically significant thoracic and intra-thoracic injury with very high sensitivity. Incorporation of these DIs into trauma evaluation protocols may allow for a safe reduction of approximately 25%–37% of non-diagnostic chest CTs, thereby reducing costs and avoiding radiation exposure in the disproportionately young trauma population.

## Supporting Information

S1 STARD STARD checklist. (DOCX)

S1 Appendix Criteria definitions. The criteria are defined for purposes of clarity and to ensure consistent data collection. They do not represent recommendations for patient evaluation.
(DOC)

## Abbreviations:

CT: computed tomography
CXR: chest X-ray
DI: decision instrument
ED: emergency department
eFAST: extended focused assessment with sonography in trauma
FAST: focused assessment with sonography in trauma
IQR: interquartile range
NPV: negative predictive value
