# Supplementary material for: Derivation and Validation of Two Decision Instruments for Selective Chest CT in Blunt Trauma: A Multicenter Prospective Observational Study (NEXUS Chest CT)
Source: PLoS Med. 2015 Oct 6;12(10):e1001883. doi: 10.1371/journal.pmed.1001883 (PMC4595216; doi:10.1371/journal.pmed.1001883)
Supplement: S1 Appendix — The criteria are defined for purposes of clarity and to ensure consistent data collection. They do not represent recommendations for patient evaluation. (DOC) [file pmed.1001883.s002.doc]

**Criteria Definitions**

The following terms are defined for purposes of clarity and to ensure consistent data collection. They do not represent recommendations for patient evaluation.

***Rapid deceleration mechanism*** refers to mechanism of blunt trauma that exerts rapid deceleration force on the patient; only 1) Fall from a height > 20 feet and 2) Motor vehicle accident at speeds > 40 MPH with sudden deceleration qualify. (**Not all > 40 MPH accidents qualify—there must be sudden deceleration. For example a 45 MPH collision with a wall or pole would qualify but a 50 MPH sideswipe or rollover MVA may not exert sudden deceleration and therefore may not qualify).** The response “unknown” is only to be used if there is no means to determine yes or no (cases in which no history is available from the patient, paramedics or others).

**The following 3 criteria are to be determined in the same manner as the NEXUS C Spine Criteria:**

***Altered mental status/abnormal alertness*** refers to a state in which the patient is not alert or not able to appropriately respond to yes/no questions. Examples of patients who would qualify as having AMS/ abnormal alertness would be those who have a Glasgow Coma Scale of 14 or less; disorientation to person, place, time or events; or delayed or inappropriate response to external stimulus.

***Distracting painful injury*** includes any condition thought by the clinician to be producing sufficient pain to significantly distract the patient from a second injury. Examples may include, but are not limited to the following: (1) long bone fractures, (2) visceral injuries requiring surgical consultation, (3) large lacerations, de-gloving injuries or crush injuries, (4) large burns, (5) spine fractures, (6) spinal cord injuries, and (7) any other injury producing acute functional impairment.

***Intoxication*** includes: a) history of intoxication or significant, recent intoxicating ingestion is provided by a patient or observer; b) test of bodily secretions (blood, urine, saliva, breath) is positive for drugs or alcohol; c) patient has physical evidence suggesting intoxication (odor of alcohol, slurred speech, ataxia, dysmetria or other cerebellar findings), or behavior consistent with intoxication and unexplained by medical or psychiatric illness.

***Chest Pain*** refers simply to the patient’s answer to the question “Do you have any chest pain?” No distinction will be made between pleuritic, positional, or other qualities of the pain. If the patient is unable to answer this question, it should be noted as *cannot determine.*

***Sternal tenderness*** refers to tenderness on palpation of the sternum.

***Chest wall tenderness to palpation*** *(CWT)* is tenderness to palpation of any part of the chest wall or thorax (boundaries defined as the upper and lower costal margins circumferentially). The physician should palpate the **anterior, posterior, and axillary** portions of the thorax. **Isolated clavicular tenderness does not qualify as CWT. Isolated sternal, scapula or thoracic spine tenderness should NOT be classified as chest wall tenderness, but rather as sternal, scapula, or thoracic spine tenderness, respectively.**

***Thoracic spine tenderness*** refers to tenderness on palpation of thoracic spine (anywhere from T1 to T12).

***Scapular tenderness*** refers to tenderness on palpation of either scapula.

***Abnormal peritoneal, pleural or pericardial fluid on FAST exam***: refers to fluid that you would deem to be abnormal (not physiologic) that would qualify as a positive FAST exam.

***Pneumothorax on e-FAST*** refers to abnormal lung sliding or other findings that you would deem to be positive for pneumothorax on e-Fast exam.
